# Tissue Culture as a Source of Replicates in Nonmodel Plants: Variation in Cold Response in *Arabidopsis lyrata* ssp. *petraea*

**DOI:** 10.1534/g3.116.034314

**Published:** 2016-10-11

**Authors:** Tanaka Kenta, Jessica E. M. Edwards, Roger K. Butlin, Terry Burke, W. Paul Quick, Peter Urwin, Matthew P. Davey

**Affiliations:** * Department of Animal & Plant Sciences, University of Sheffield, S10 2TN, UK; † Centre for Plant Sciences, Institute of Integrative and Comparative Biology, University of Leeds, LS2 9JT, UK

**Keywords:** reaction norm, stress tolerance, genetic architecture, genetic basis, adaptive variation

## Abstract

While genotype–environment interaction is increasingly receiving attention by ecologists and evolutionary biologists, such studies need genetically homogeneous replicates—a challenging hurdle in outcrossing plants. This could be potentially overcome by using tissue culture techniques. However, plants regenerated from tissue culture may show aberrant phenotypes and “somaclonal” variation. Here, we examined somaclonal variation due to tissue culturing using the response to cold treatment of photosynthetic efficiency (chlorophyll fluorescence measurements for *F_v_/F_m_*, *F_v_′/F_m_′*, and Φ_PSII_, representing maximum efficiency of photosynthesis for dark- and light-adapted leaves, and the actual electron transport operating efficiency, respectively, which are reliable indicators of photoinhibition and damage to the photosynthetic electron transport system). We compared this to variation among half-sibling seedlings from three different families of *Arabidopsis lyrata* ssp. *petraea*. Somaclonal variation was limited, and we could detect within-family variation in change in chlorophyll fluorescence due to cold shock successfully with the help of tissue-culture derived replicates. Icelandic and Norwegian families exhibited higher chlorophyll fluorescence, suggesting higher performance after cold shock, than a Swedish family. Although the main effect of tissue culture on *F_v_/F_m_*, *F_v_′/F_m_′*, and Φ_PSII_ was small, there were significant interactions between tissue culture and family, suggesting that the effect of tissue culture is genotype-specific. Tissue-cultured plantlets were less affected by cold treatment than seedlings, but to a different extent in each family. These interactive effects, however, were comparable to, or much smaller than the single effect of family. These results suggest that tissue culture is a useful method for obtaining genetically homogenous replicates for studying genotype–environment interaction related to adaptively-relevant phenotypes, such as cold response, in nonmodel outcrossing plants.

Genotype–environment interaction effects on a phenotype, or variation in reaction norms, may modulate natural selection (Wright 1931; Sultan 1987). The genetic basis of genotype–environment interaction is increasingly receiving attention (El-Soda *et al.* 2014; Yap *et al.* 2011); however, such advances have been concentrated in inbreeding organisms such as *Arabidopsis thaliana* (*e.g.*, Bloomer *et al.* 2014; Sasaki *et al.* 2015; Stratton 1998; El-Soda *et al.* 2014) and *Caenorhabditis elegans* (Gutteling *et al.* 2007), because genetically isogenic individuals derived by repeated inbreeding permit a given genotype to be exactly repeated in multiple environments. Recently, the wild relatives of model organisms have increasingly been exploited by evolutionary biologists to understand adaptation and speciation (Mitchell-Olds 2001; Clauss and Koch 2006). However, one disadvantage of nonmodel plants with outcrossing mating systems is that they cannot usually be exploited to produce the genetically homogeneous or inbred recombinant lines that enable researchers to study the reaction norms of single genotypes in multiple environments (Dorn *et al.* 2000)or to map novel QTL in previously genotyped lines (Alonso-Blanco *et al.* 2005). This disadvantage could be compensated for by using cutting techniques to produce multiple clones from single genotypes (Sultan and Bazzaz 1993; Waitt and Levin 1993; Wu 1998). This method is applicable only to plants capable of vegetative propagation, and it also needs relatively large plant bodies to produce many replicate clones. Another technique applicable to a wider range of plants with relatively small starting plant material is tissue culture (George and Sherrington 1984). However, tissue culture has been exploited only rarely for studies on the genetic basis of genotype–environment interaction, and the few existing studies (Glock and Gregorius 1986; Glock 1989) focused only on callus characteristics as target phenotypes. One potential issue that should be considered carefully is that tissue-culture derived microshoots can express phenotypic, “somaclonal” variation (Larkin and Scowcroft 1981), or may sometimes show aberrant morphology and physiology *in vitro* (Joyce *et al.* 2003). This somaclonal variation resembles that induced by physical mutagens, with elevated levels of chromosome breakage and rearrangement, polyploidy, aneuploidy, transposon activation, and point mutation (D’Amato and Bayliss 1985). Therefore, with a view to exploiting the techniques of tissue culturing more widely in studies of genotype–environment interaction in outcrossing plants, it is necessary to extend our knowledge on how propagation by tissue culture generates variation in phenotypes that are relevant to adaptation in natural environments, compared to other sources of genetically related replicates such as outbred siblings.

Key plant properties that have attracted marked attention in the field of adaptation to various environments are stress tolerances (*e.g.*, Quesada *et al.* 2002; Kwon *et al.* 2007; Zhang *et al.* 2004; Zhen and Ungerer 2008; Steponkus *et al.* 1998; Hong and Vierling 2000; Lexer *et al.* 2003). One trait that can be used to indicate tolerance against various physical stressors in plants is photosynthetic performance. Photosystem II (PSII) activity is sensitive to both biotic and abiotic factors (Murchie and Lawson 2013). Chlorophyll fluorescence can be used to determine the maximum efficiency with which light absorbed by pigments of photosystem II (PSII) is used to drive photochemistry in dark- (*F_v_/F_m_*) or light- (*F_v_′/F_m_′*) adapted material and the operating efficiency of PSII (Φ_PSII_). It is a reliable indicator of photoinhibition and damage to the photosynthetic electron transport system (Quick and Stitt 1989; Maxwell and Johnson 2000). Changes in chlorophyll fluorescence have been used in *Arabidopsis thaliana* to quantify tolerance to cold and freezing temperatures. Ehlert and Hincha (2008) showed that chlorophyll fluorescence imaging detected difference in freezing tolerance between two *A. thaliana* lineages, both before and after cold acclimation. Mishra *et al.* (2014) applied chlorophyll fluorescence imaging for nine *A. thaliana* lineages under cold and freezing temperature, and suggested that freezing tolerance of lineages could be screened by chlorophyll fluorescence under cold (4°) condition without exposing plants to subzero temperature. Chlorophyll fluorescence has also been used to study tolerance to drought (Woo *et al.* 2008; McAusland *et al.* 2013; Bresson *et al.* 2015), and salt and heavy-metal stress (Yuan *et al.* 2013), in *A. thaliana*, as well as in various other plants for tolerance or response to cold and freezing temperatures (Baldi *et al.* 2011; Medeiros *et al.* 2012; Xie *et al.* 2015; Khanal *et al.* 2015; Heo *et al.* 2014), drought (Jansen *et al.* 2009), and salt (Yuan *et al.* 2013). If variation in chlorophyll fluorescence can be properly estimated using tissue-culture derived clones, therefore, this method would enhance studies in genotype–environment interaction for stress tolerance in outcrossing plants.

To this end, we have studied change in chlorophyll fluorescence following cold shock in a wild relative of a model plant species. *A. lyrata* ssp. *petraea* is a close relative of the model species *A. thaliana*, but with a different ecology, life history and population genetics (Charlesworth *et al.* 2003; Davey *et al.* 2008, 2009; Kuittinen *et al.* 2008; Kunin *et al.* 2009). While *A. thaliana* is mainly selfing, with a low level of genetic diversity within a population, *A. lyrata* ssp. *petraea* is outcrossing, with a high level of genetic diversity even within a population (Clauss and Mitchell-Olds 2006; Kunin *et al.* 2009; Heidel *et al.* 2006; Schierup *et al.* 2008). Further studies on genetic and phenotypic variation in spatially distinct individuals and in closely related plants will clarify whether or not locally advantageous alleles are fixed, and if local populations are in evolutionary equilibrium, and are thus important in our understanding of the evolutionary responses to environmental change. Distinguishing phenotypic variation among closely related individuals from measurement errors is difficult; however, this becomes possible if we can quantify the error within the same genotype using tissue-cultured clones.

In this study, we measured the chlorophyll fluorescence parameters *F_v_/F_m_*, *F_v_′/F_m_′*, and Φ_PSII_ before and after cold shock, as an index of cold response, for seedlings from three families from geographically isolated populations of *A. lyrata* ssp. *petraea*, and tissue-cultured plantlets derived from several genotypes (seeds) in each of those families (Table 1). In order to evaluate the usefulness of tissue culture for obtaining genetically homogenous replicates and to assess how much adaptively relevant variation exists within the species, we tested whether (i) among-genotype phenotypic variation could be detected with the help of replication of tissue cultured plantlets; (ii) somaclonal variation would remain in the range of other components of variation, such as within-family variation of seedlings; (iii) phenotypic variation in putatively adaptive traits would exist between families; and (iv) tissue-culturing affected these measurements of chlorophyll fluorescence.

**Table 1:** Numbers of plants and blocks in each family (Ardal, Notsand, and Sandfell)

	Genotype 1	Genotype 2	Genotype 3	Half Sibs
Ardal				
Number of plants	33	36	–	40
Number of blocks	4	4	–	10
Plants/block (min – max)	6–9	9–9	–	4–4
Notsand				
Number of plants	13	31	–	40
Number of blocks	2	4	–	10
Plants/block (min – max)	5–8	4–9	–	4–4
Sandfell				
Number of plants	45	28	23	28
Number of blocks	5	4	3	4
Plants/block (min – max)	9–9	5–9	5–9	5–8

Plants were either seedlings in a half-sibling family, or tissue-cultured clonal plantlets from genotypes derived from a seed from each family. Block refers to the groups of plantlets from each genotype, or groups of seedlings from the same family for half-sibling families, that were treated and measured at the same time.

## Materials and Methods

### Plants

Seeds of *A. l*. *petraea* were collected from geographically separated populations in Ardal (Norway) (61°19′25″N, 7°50′00″E, alt. 63 m), Notsand (Sweden) (62°36′31″N, 18°03′37″E, alt. 3 m), and Sandfell (Iceland) (64°04′14″N, 21°41′06″E, alt. 123 m). No specific permits were required for the seed collection for this study because these locations were not privately owned or protected in any way, and because the species was not protected in these countries. The species is a perennial herb, maintaining leaves throughout the year. We used a family of seeds that were at least half-siblings, from one mother plant in each population. We grew 28–40 seedlings per family, and, in each case, derived 44–69 tissue-cultured plantlets from two to three seeds (one genotype = cloned plantlets from one seed) of each family.

### Tissue culture

Seeds were sterilized in 10% commercial bleach for 20 min, washed in sterile water and stored at 4° overnight. The seeds were then placed onto 50% strength Murashige and Skoog (MS) medium (Melford Laboratories Ltd, Ipswich, UK), pH 5.7, supplemented with 1% sucrose, 5 mg/l silver thiosulfate, and solidified with 1% plant agar (Melford Laboratories). The agar plates were held vertically, allowing for maximum recovery of root tissue. After 4 wk, the root systems were excised and placed intact onto Callus Induction Medium (CIM) (Clarke *et al.* 1992) solidified with 0.55% plant agar. Plates were incubated at 23° for 3 d, then the roots were cut into 5 mm lengths and placed in bundles on fresh CIM plates that were further incubated at 20° for 2–3 d. The root sections from each plant were resuspended in 10 ml molten Shoot Overlay Medium (SOM) (Clarke *et al.* 1992), solidified with 0.8% low gelling-temperature agarose, and poured over a single 90 mm plate of Shoot Induction Medium (SIM) (Clarke *et al.* 1992) solidified with 0.55% plant agar and lacking antibiotics. The plates were incubated at 20° under a 16-hr day length. Once shoots started to form from the calli, they were transferred to 50% strength MS medium, pH 5.7, supplemented with 1% sucrose and solidified with 0.55% plant agar, such that each plate contained nine clones of the same genotype. A total of four to nine plantlets survived per plate. Each plate was treated as a block in the following experiment.

### Seedling growth

Seeds were sown in Levington M3 compost within individual plug trays. Families were randomized within each tray, and trays were repositioned randomly every other day. Plants were watered from the base of the pot as required with reverse-osmosis (RO) purified water. No additional nutrients were added to the soil or water. Plants were established to the six- to eight-leaf stage in controlled-environment growth cabinets (Conviron Controlled Environments Limited, Canada) set to a 12/12 hr day/night cycle, 20/15° d/night, 70% humidity; atmospheric CO_2_ concentration was 400 ppm, and photosynthetically active radiation 250 µmol m^−2^ s^−1^. Chlorophyll fluorescence measurements were taken just prior to, and after, a 24-hr cold treatment in which plants were exposed to the same conditions as above, apart from the temperature being decreased to 3°. Four to eight seedlings from the same family that were tested together were treated as a block in the subsequent experiment.

### Chlorophyll fluorescence

Precold and postcold treatment measurements of chlorophyll fluorescence were obtained using a chlorophyll fluorescence imager using Fluorimager software (Technologica Ltd., Colchester, UK). Each block of plants was dark-adapted for at least 15 min before the maximum efficiency of photosystem II (*F_v_/F_m_*) was measured to a blue light pulse at 3000 µmol m^−2^ s^−1^ for 200 msec. Following this pulse, the plants were exposed to an actinic light of 150 µmol m^−2^ s^−1^ for 6 min, followed by pulses of 3000 µmol m^−2^ s^−1^ for 200 msec to obtain measures of maximum efficiency of photosystem II (*F_v_′/F_m_′*) of light-adapted plant material and the operating efficiency of photosystem II (Φ_PSII_) in light-adapted plant material. Mean values of *F_v_/F_m_*, *F_v_′/F_m_′*, and Φ_PSII_ for each plant were taken from the image of each whole plant.

### Statistical analyses

To examine the relative importance of among-family and among-genotype variation in cold response, we used nested ANOVA to partition the total variance in the difference in each chlorophyll fluorescence measurement (*F_v_/F_m_*, *Fv′/Fm′*, or Φ_PSII_) induced by cold shock:

P∼Family/Genotype/Block

for tissue culture material, or

P∼Family/Block

for seedlings, where *P* is the difference in each type of chlorophyll fluorescence for a plant individual between two measurements (*i.e.*, value after cold shock minus that before cold shock), the “/” symbol implies nesting and terms were fitted as fixed effects. Variance in *P* was partitioned such that:

Total variance=V(Family)+V(Genotype)+V(Block)

for tissue culture material, or

Total variance=V(Family)+V(Block)

for seedlings.

To evaluate variation in each natural and tissue-cultured condition, we did this analysis separately for the tissue-cultured plants and seedlings. We conducted these variance component analyses using the varcomp function in the ape library, and the lme function in *R* 2.8.0 (*R* Development Core Team 2008).

We tested whether variance in the change of *F_v_/F_m_*, *Fv′/Fm′*, or Φ_PSII_ due to cold shock among tissue-culture derived plantlets within each genotype was different from that in seedlings of half-siblings of the same family using Bartlett tests. Because the number of blocks differed between seedlings and tissue-cultured plantlets (Table 1), we checked first whether the difference in the number of blocks affected the variance, by resampling all possible combinations of four blocks from the 10 blocks of half-siblings in Ardal and Notsand. Reducing block number changed the original variance for 10 blocks only < ± 3% without systematic bias.

Finally, we evaluated the effect of several factors on each type of chlorophyll fluorescence measurement before and after cold treatment. We constructed the following linear mixed-effect model, in which plant individual was treated as a random effect:

CF=I|B/P+C+T+F+C×T+T×F+C×F+C×T×F

where CF was a single measurement of either *F_v_/F_m_*, *Fv′/Fm′*, or Φ_PSII_, and I|B/P was the intercept with random effects of block, and individual plant nested in each block, C was a categorical variable of cold shock (cold-shocked or not), T was a categorical variable of tissue culture (tissue-cultured or not), and F was a categorical variable of family (three families), followed by the interaction terms among those variables. The effect of each term was estimated by the lme function using the statistical software *R* 2.8.0 (R Development Core Team 2008). Akaike’s Information Criterion (AIC) was compared between the full model and a model lacking each term in a stepwise manner, and the best model with the lowest AIC was selected, followed by testing the significance of each selected parameter using the Wald test.

### Data availability

The authors state that all data necessary for confirming the conclusions presented in the article are represented fully within the article. All phenotypic data are available in Dryad Digital Repository: http://dx.doi.org/10.5061/dryad.9gs8k.

## Results and Discussion

### Variance components in cold-response of Fv/Fm, Fv′/Fm′, and Φ_PSII_

In the seedlings, the changes in *F_v_/F_m_*, *Fv′/Fm′*, or Φ_PSII_ following cold treatment varied significantly among families, explaining 4.9–9.1% of the total variance (Table 2). For the tissue-cultured plantlets, the change in those indices following cold treatment did not vary significantly among families, but did vary significantly among genotypes within family, this component explaining 8.5–31.5% of the total variance. The within-block (error) variance component for tissue-cultured plantlets was 61.7–81.8%, and tended to be smaller than this component for seedlings (89.1–92.2%).

**Table 2:** Analysis of variance for change in *F_v_/F_m_*, *F_v_′/F_m_′* and Φ_PSII_ by cold treatment for nontissue-cultured seedlings and tissue-cultured plantlets

	Seedlings						Tissue Cultures					
	Df	Sum Sq	Mean Sq	*F*	*P*	Variance Component (%)	Df	Sum Sq	Mean Sq	*F*	*P*	Variance Component (%)
*Fv/Fm*												
Family	2	0.027	0.013	2.84	0.081	4.9	2	0.002	0.001	0.06	0.946	0.0
Genotype							4	0.080	0.020	11.52	0.000	31.5
Block	21	0.098	0.005	1.11	0.351	6.1	19	0.033	0.002	1.91	0.016	6.8
Error	84	0.353	0.004			89.1	183	0.167	0.001			61.7
*F_v_′/F_m_′*												
Family	2	0.081	0.041	10.01	0.001	7.8		0.005	0.002	0.24	0.798	0.0
Genotype							4	0.041	0.010	3.27	0.034	10.9
Block	21	0.085	0.004	0.33	0.997	0.0	19	0.059	0.003	2.54	0.001	14.1
Error	84	1.048	0.012			92.2	183	0.225	0.001			74.9
Ф_PSII_												
Family	2	0.026	0.013	8.44	0.002	9.1	2	0.044	0.022	2.81	0.173	7.7
Genotype							4	0.031	0.008	3.37	0.030	8.5
Block	21	0.032	0.002	0.45	0.978	0.0	19	0.044	0.002	1.23	0.241	2.0
Error	84	0.282	0.003			90.9	183	0.349	0.002			81.8

Family and Block refer to variation among families and among blocks within families, respectively. Each Block was a group of seedlings from the same family for Seedling or group of plantlets from the same genotype for Tissue cultures. Error refers to variation among plants within blocks.

### Evaluation of somaclonal variation in comparison to within-family variation

**Figure 1:**
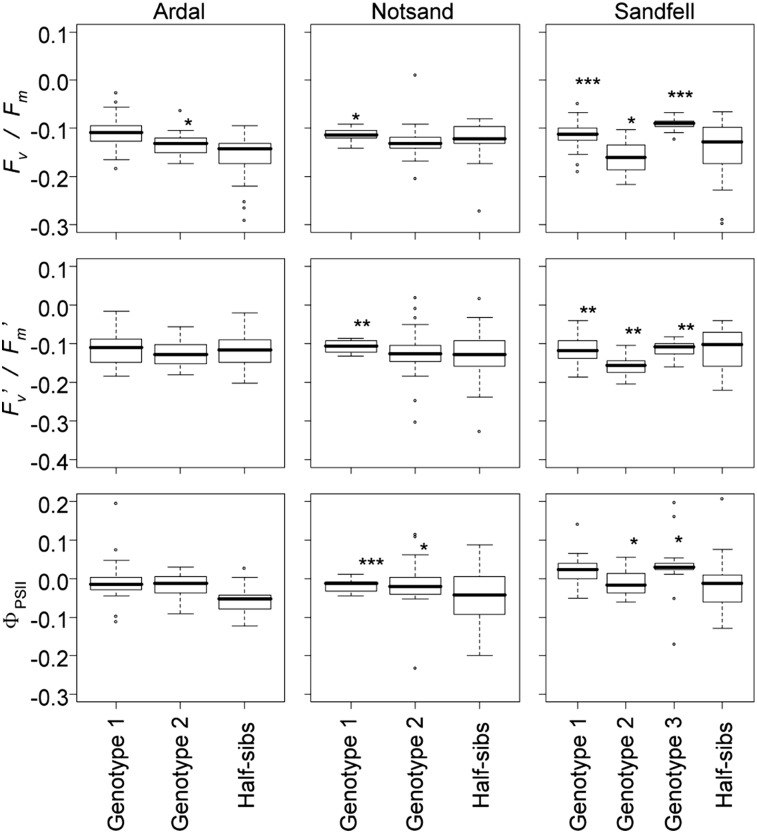
Change in chlorophyll fluorescence (*F_v_/F_m_*, *F_v_′/F_m_′*, and Φ_PSII_) in seedlings or plantlets originating from Norway (Ardel), Sweden (Notsand), and Iceland (Sandfell) after cold-treatment (values after shock – those before shock). * = *P* *<* 0.05, ** = *P* *<* 0.01, and *** = *P* *<* 0.001 (Bartlett test) indicate a significantly lower variance of the genotype than among half-siblings in the same family. Three *F_v_/F_m_* values (0.340, 0.375, and 0.592), and an *F_v_′/F_m_′* value (0.354) in Sandfell half-siblings were out of the vertical ranges shown, but were included in the statistical tests.

Variances in the change of *F_v_/F_m_*, *Fv′/Fm′*, or Φ_PSII_ among clones within genotype were clearly smaller than those among half-siblings of the same family in the Sandfell family. Most genotypes had significantly smaller variances in *F_v_/F_m_*, *F_v_′/F_m_′*, and Φ_PSII_ than half-sibs as shown by the Bartlett test (Figure 1). Similar patterns were observed in Notsand and Ardal. No studied genotype had larger variance among clones than the variance among half-siblings in any family.

### Effects of cold shock, tissue culturing, and family on Fv/Fm, Fv′/Fm′, and Φ_PSII_

All single effects of cold shock, tissue culture and family and all possible interaction combinations among them affected *F_v_/F_m_* and *F_v_′/F_m_′*, and all such effects except the three-way interaction between cold shock, tissue culture and family affected Φ_PSII_, according to the best model (Table 3) based on Akaike’s Information Criterion (AIC). Cold shock and family were the strongest single effects. The interaction between these two factors was also found to change all three measurements of chlorophyll fluorescence, indicating that the effect of cold shock depended on family. The effect of tissue culture was relatively small and not significant for any of the chlorophyll fluorescence measures. We found substantial interactions between tissue culture and family, and interactions among cold shock, tissue culture, and family, indicating that the effect of tissue culture varied among families.

**Table 3:** The best linear mixed models for *F_v_/F_m_*, *F_v_′/F_m_′* and Φ_PSII_, based on AIC

	Estimates	SE	DF	*t*	*P*
*Fv/Fm*					
Intercept	0.787	0.011	311	71.3	<0.001
Cold shock	−0.122	0.008	311	−15.9	<0.001
Tissue culture	−0.017	0.015	302	−1.1	0.252
Fam A	−0.026	0.015	302	−1.7	0.093
Fam S	−0.091	0.017	302	−5.4	<0.001
Cold shock × Tissue culture	−0.007	0.011	311	−0.7	0.506
Cold shock × Fam A	−0.035	0.011	311	−3.2	0.002
Cold shock × Fam S	−0.007	0.012	311	−0.5	0.584
Tissue culture × Fam A	0.029	0.020	302	1.5	0.147
Tissue culture × Fam S	0.082	0.021	302	3.9	<0.001
Cold shock × Tissue culture × Fam A	0.043	0.014	311	3.0	0.003
Cold shock × Tissue culture × Fam S	0.015	0.015	311	1.0	0.327
*Fv′/Fm′*					
Intercept	0.695	0.014	311	50.9	<0.001
Cold shock	−0.131	0.011	311	−12.1	<0.001
Tissue culture	−0.019	0.019	302	−1.0	0.304
Fam A	−0.050	0.019	302	−2.6	0.009
Fam S	−0.167	0.021	302	−7.9	<0.001
Cold shock × Tissue culture	0.011	0.015	311	0.8	0.446
Cold shock × Fam A	0.015	0.015	311	0.9	0.345
Cold shock × Fam S	0.068	0.017	311	4.0	<0.001
Tissue culture × Fam A	0.070	0.025	302	2.8	0.006
Tissue culture × Fam S	0.181	0.026	302	6.9	<0.001
Cold shock × Tissue culture × Fam A	−0.013	0.020	311	−0.7	0.514
Cold shock × Tissue culture × Fam S	−0.077	0.021	311	−3.7	<0.001
Ф_PSII_					
Intercept	0.403	0.012	313	34.2	<0.001
Cold shock	−0.047	0.006	313	−7.7	<0.001
Tissue culture	−0.027	0.016	302	−1.7	0.090
Fam A	−0.029	0.016	302	−1.8	0.081
Fam S	−0.086	0.018	302	−4.7	<0.001
Cold shock × Tissue culture	0.034	0.006	313	5.8	<0.001
Cold shock × Fam A	−0.004	0.007	313	−0.5	0.610
Cold shock × Fam S	0.028	0.007	313	3.9	<0.001
Tissue culture × Fam A	−0.005	0.021	302	−0.2	0.822
Tissue culture × Fam S	0.043	0.022	302	2.0	0.051

Fam A and Fam S refer to families Ardal and Sandfell, respectively. Intercepts represent the combination of background conditions, *i.e.*, not cold shocked, not tissue cultured, and family Notsand. All effects are for family Notsand unless another family name was shown. Effects for the other families are shown as differences from the background effect of family Notsand.

### Among-genotype variance

We were able to test for among-genotype variance using replicates generated by tissue culture within genotypes, and we detected such variance in *F_v_/F_m_*, *F_v_′/F_m_′*, and Φ_PSII_ measurements (Table 2). On the other hand, we showed significant but low somaclonal variation. The within-block (error) variance component for tissue-cultured plantlets was relatively small compared to that for nontissue-cultured seedlings (Table 2). The Bartlett tests showed that somaclonal variation was smaller than, or at least remained within the range of, the within-family variance, which is the smallest naturally observed component of variation in the hierarchy of genetic structure (Figure 1). In *A. thaliana*, studies of natural variation have focused mainly on between-population variation (*e.g.*, Shindo *et al.* 2007). In contrast, *A. lyrata* has substantial within-population variation, for example, in the composition of glucosinolates (Clauss *et al.* 2006) or self-incompatibility genes (Schierup *et al.* 2008). In this paper, we showed that there is within-family as well as among-family, and thus among-population, genetic variation in *A. lyrata* ssp. *petraea*. Within-family genetic variance was relatively large in Sandfell (Iceland). The observed within-family genetic variances in putatively adaptive traits highlight the wide potential for evolutionary adaptation of the species, and further validate the usefulness of relatives of model organisms in evolutionary biology (Mitchell-Olds 2001; Clauss and Koch 2006).

### Among-family variance

There was significant or marginally significant among-family variance in the change of *F_v_/F_m_*, *F_v_′/F_m_′*, and Φ_PSII_ values following cold treatment for seedlings (Table 2). We used different growth chambers for plant growth and for cold shock, and therefore light condition for cold shock inevitably differed from that for growth. Light and temperature are difficult to disassociate in such a study system, and both the single effect of cold treatment, and the light–temperature interaction, can be involved in the effect of cold shock. In *A. thaliana*, the change in chlorophyll fluorescence from before to after cold shock correlates with tolerance to subzero temperatures measured by electrolyte leakage, and, therefore, this is regarded as an indicator of cold tolerance or response (Ehlert and Hincha 2008; Mishra *et al.* 2014). Therefore, our result also represents evidence for among-family (thus possibly among-population) variance in cold response.

### Effects of tissue culturing

We detected genotype-specific effects of tissue culture on *F_v_/F_m_*, *F_v_′/F_m_′*, and Φ_PSII_ (Table 3, and Supplemental Material, Table S1). This is consistent with a previous report of a genotype-specific effects on callus characteristics (Glock and Gregorius 1986; Glock 1989). The three measured parameters of chlorophyll fluorescence all decreased after the cold shock (the effects of cold shock in Table 3 are all negative for *F_v_/F_m_*, *F_v_′/F_m_′*, and Φ_PSII_), indicating a decrease in photosystem II activity, as reported in previous studies (Finazzi *et al.* 2006). A positive effect of interaction between tissue culture and cold shock for Φ_PSII_ suggests that tissue-cultured plants were less affected by cold shock than seedlings, and an interaction between tissue culture, cold shock, and family suggests that the extent to which tissue-cultured plants were less affected by cold shock differed among families. Any differences among families in traits related to responses to the tissue-culture environment, including root-cutting, callus formation, and growth on medium, might explain these observed interactions between tissue culture and family. This finding is consistent with the report that somaclonal variation is genotype-dependent, and influenced by both the explant source and the tissue-culture protocol (George and Sherrington 1984), and with a recent study showing that the effect of tissue culture on somatic mutations depended on genotype (Zhang *et al.* 2010). The effects of tissue culture–genotype interaction, however, were comparable to, or much smaller than, the single effect of family (Table 3), indicating that such interactions would not mask the single effect of genotype. The interaction effect between tissue culture and family was much smaller in Φ_PSII_ than in *F_v_/F_m_* or *F_v_′/F_m_′* [the ranges between maximum and minimum estimates were 0.043 − (−0.005) = 0.048, 0.082 − 0 = 0.082 and 0.181− 0 = 0.181, respectively; Table 3]. An interaction between cold shock, tissue culture, and family was detected only in *F_v_/F_m_* and *F_v_′/F_m_′*. Also, the relative impact of among-genotype variance was smaller for Φ_PSII_ (8.5% of the total variance, Table 2) than for *F_v_/F_m_* (31.5%) or *F_v_′/F_m_′* (10.9%). These results imply that, although the maximum efficiencies of photosynthesis for dark- (*F_v_/F_m_*) and light-adapted leaves (*F_v_′/F_m_′*) were affected by tissue culturing in genotype-specific ways, the actual electron transport operating efficiency (Φ_PSII_) was less affected by tissue culture.

### Conclusion

Overall, we successfully detected among-genotype variance, with low somaclonal variation, indicating that the advantage of tissue culturing in generating genetically isogenic replicates exceeded its disadvantage in amplifying somaclonal variation in our study system. We detected interaction effects of tissue culture with genotype for a putatively adaptive trait, cold response; however, such variation would not mask the single effect of genotype. Therefore, although one should consider effects of tissue culturing carefully when interpreting any results relying on the technique, tissue culturing is a useful method for obtaining genetically homogenous replicates in this, and probably other, nonmodel organisms. It can provide critical additional power when studying phenotypes such as cold response related to adaptation in natural environments, the variation in the phenotypes among families or populations, the reaction norms of a genotype, or the QTL accounting for phenotypes.

## Supplementary Material

Supplemental Material
