# Supplementary material for: Tissue Culture as a Source of Replicates in Nonmodel Plants: Variation in Cold Response in Arabidopsis lyrata ssp. petraea
Source: G3 (Bethesda). 2016 Oct 11;6(12):3817–23. doi: 10.1534/g3.116.034314 (PMC5144953; doi:10.1534/g3.116.034314)
Supplement: Supplemental Material [file supp_6_12_3817__index.html]

Tissue Culture as a Source of Replicates in Non-model Plants: Variation in Cold Response in Arabidopsis lyrata ssp. petraea — Tissue Culture as a Source of Replicates in Nonmodel Plants: Variation in Cold Response in Arabidopsis lyrata ssp. petraea — Supplemental Material 

# Tissue Culture as a Source of Replicates in Nonmodel Plants: Variation in Cold Response in *Arabidopsis lyrata* ssp. *petraea*

## Supplemental Material for Kenta, *et al*, 2016

**Files in this Data Supplement:**

- Table S1 - Akaike's Information Criterion (AIC) of each examined linear mixed models for *Fv/Fm, Fv'/Fm'* and ΦPSII with effects specified by "+". (.pdf, 233 KB)
