# Supplementary material for: Tissue Culture as a Source of Replicates in Nonmodel Plants: Variation in Cold Response in Arabidopsis lyrata ssp. petraea
Source: G3 (Bethesda). 2016 Oct 11;6(12):3817–23. doi: 10.1534/g3.116.034314 (PMC5144953; doi:10.1534/g3.116.034314)
Supplement: Supplemental Material [file supp_g3.116.034314_TableS1.pdf]

Table S1. Akaike's Information Criterion (AIC) of each examined linear mixed models for F_v/F_m, F_v'/F_m' and Φ_{PSII} with effects specified by "+". The best model (top) with the lowest AIC was selected for each of F_v/F_m, F_v'/F_m' and Φ_{PSII} and their details are shown in Table 3. C: cold shock (cold-shocked or not), T: tissue culture (tissue-cultured or not), and F: family. "×" indicates interaction effects between two or three variables. Delta indicates difference in AIC from the best model.

	C	T	F	C×T	C×F	T×F	C×T×F	AIC	Delta
Fv/Fm									
	+	+	+	+	+	+	+	-1846.6	0.0
	+	+	+	+		+		-1842.3	4.3
	+	+	+	+	+	+		-1841.1	5.5
	+	+	+			+		-1838.6	8.0
	+	+	+		+	+		-1837.8	8.7
	+	+	+	+				-1826.0	20.6
	+	+	+	+	+			-1824.7	21.8
	+	+	+					-1822.3	24.3
	+	+	+		+			-1821.5	25.1
	+	+		+				-1814.8	31.7
	+		+					-1812.5	34.1
	+		+		+			-1811.7	34.9
	+	+						-1811.1	35.4
	+							-1805.6	40.9
		+	+			+		-1192.8	653.8
		+	+					-1177.7	668.8
			+					-1168.5	678.0
		+						-1167.2	679.4
								-1162.0	684.6
Fv'/Fm'									
	+	+	+	+	+	+	+	-1503.2	0.0
	+	+	+	+		+		-1493.0	10.2
	+	+	+			+		-1491.6	11.6
	+	+	+	+	+	+		-1491.5	11.7
	+	+	+		+	+		-1488.9	14.3
	+	+	+	+				-1463.2	40.0
	+	+	+					-1461.8	41.4
	+	+	+	+	+			-1461.7	41.5
	+	+	+		+			-1459.1	44.1
	+	+		+				-1454.5	48.7
	+	+						-1453.1	50.1
	+		+					-1432.4	70.9

	+							-1430.2	73.0
	+		+		+			-1429.7	73.5
		+	+			+		-1069.7	433.5
		+	+					-1039.9	463.4
		+						-1031.2	472.1
			+					-1010.5	492.8
								-1008.3	495.0
Φ_{PSII}									
	+	+	+	+	+	+		-1768.6	0.0
	+	+	+	+	+			-1766.5	2.1
	+	+	+	+	+	+	+	-1766.3	2.2
	+	+	+	+		+		-1745.7	22.9
	+	+	+	+				-1743.6	25.0
	+	+	+		+	+		-1738.2	30.4
	+		+		+			-1738.0	30.5
	+	+	+		+			-1736.1	32.5
	+	+		+				-1730.7	37.8
	+	+	+			+		-1705.8	62.7
	+		+					-1705.7	62.8
	+	+	+					-1703.8	64.8
	+							-1692.6	75.9
	+	+						-1690.9	77.6
		+	+			+		-1683.9	84.7
			+					-1683.8	84.8
		+	+					-1681.8	86.7
								-1670.6	97.9
		+						-1669.0	99.6
